# Why do rural youth migrate? Evidence from Colombia and Guatemala

**DOI:** 10.3389/fsoc.2024.1439256

**Published:** 2024-08-06

**Authors:** Manuel Francisco Díaz Baca, Leonardo Moreno Lerma, Stefan Burkart, Natalia Triana Ángel

**Affiliations:** ^1^Department of International Agricultural Policy and Environmental Governance, University of Kassel, Kassel, Germany; ^2^Tropical Forages Program, International Center for Tropical Agriculture (CIAT), Cali, Colombia; ^3^Independent Researcher, Cali, Colombia

**Keywords:** rural migration, public policy, climate change mitigation, climate change adaptation, sustainability, competitiveness

## Abstract

Migration, from rural to urban settings is a common phenomenon in Latin America, due to social, economic, political, and other factors. Young people in search of economic and educational opportunities, financial, and social stability, have been migrating to larger urban centers, thus crafting important shifts in rural labor, generational transfer, and domestic economies. Through a systematic literature review of scientific literature, and documents from public institutions and international organizations, published between 2012 and 2022, this article addresses rural–urban migration of youth in Colombia and Guatemala’s cattle sector, particularly identifying (i) driving factors, (ii) their impacts on cattle farming, and (iii) public policies implemented to counteract prejudicial effects. Results show that unemployment, lack of educational opportunities, and insecurity are the main reasons for youth migration to cities or abroad, with Mexico, the United States, and Spain being the most common destinations. Additionally, impacts on the cattle sector include shortage of labor and a perfectible generational transfer, hindering the modernization of the industry and investments in climate change adaptation and mitigation strategies. Despite various implemented public policies, the results are partial, and the issue of accelerated youth migration remains relevant. Consequently, without more effective measures adopted by national governments, the cattle sector will lag behind its regional and international competitors, deterring the achievement of the Sustainable Development Goals. As the main contribution of the study, the analysis of migration is highlighted based on its effects on a specific economic sector and not focused on its causes, as evidenced in a wide range of literature.

## Highlights

- The origins of migration in Colombia and Guatemala are concentrated in rural areas.
- Limited labor availability in the cattle sector is aggravated by youth migration.
- Policies identify the drivers of migration and define inclusion criteria for youth.

## 1 Introduction

Human migration has been a constant from ancient to modern times (Sirbu et al., 2021), driven by macro-social, political, economic, and environmental factors (Castelli, 2018). Latin America is not exempt from this phenomenon, currently serving as both an origin and transit region for migrants (International Organization for Migration, 2020). In 2018, 1.3 million people from Latin America and the Caribbean migrated to countries in the Organization for Economic Cooperation and Development (OECD), with the United States and Spain being their main destinations (Inter-American Development Bank, 2021). Additionally, there is an intraregional migration process, as four out of every five migrants from South America reside in another country within the subregion (International Organization for Migration, 2022a).

Among the distinct types of migration, the movement from rural to urban areas stands out, particularly among young people seeking to escape precarious socio-economic conditions (Zabala, 2021). In Latin America, out of the 31 million rural youth aged 15–29, 11.9 million are unemployed, experiencing higher poverty levels compared to adults in those regions and individuals of the same age in urban areas (OECD and FAO, 2019). Academic literature indicates that this age group is more likely to migrate compared to adults, with males surpassing females in this regard (Prieto et al., 2022). While youth immigration has positive repercussions in the destination, such as rejuvenating the population, it has adverse effects on the place of origin by accelerating aging and, consequently, reducing the working-age population (García et al., 2019). One of the most affected sectors is agriculture, facing challenges like labor shortages (Rosendo et al., 2019), hampering the productivity of rural activities due to the difficulty for older farmers to adopt and finance the latest technologies (Zabala, 2021).

This research aims to address the phenomenon of youth migration and its impacts on the agricultural sector in Colombia and Guatemala. It is noteworthy that the focus is on the cattle sector due to its significance for Latin America and the Caribbean, contributing over 14% of the total agricultural production value (FAO, 2018), serving as a crucial source of income for the rural population, and generating employment for millions of people. Cattle farming is also vital for the region, aiding in achieving Sustainable Development Goals (SDGs), i.e., SDG-1 (no poverty), SDG-2 (zero hunger), SDG-6 (gender equality), SDG-8 (decent work and economic growth), SDG-10 (reduced inequalities), SDG-13 (climate action), among others (United Nations, 2018). Consequently, establishing connections between youth migration and cattle farming not only helps understand the issues affecting this population but also the negative impacts migration is having on a fundamental sector in economic, environmental, and social terms.

The selection of these countries aligns with several criteria: (i) Both rank high among countries with the most migrants in their regions: Colombia is second in South America, surpassed only by Venezuela, and Guatemala is second in Central America after El Salvador (Statista, 2023a); (ii) Colombia designed the Sustainable Cattle Policy, while Guatemala implemented the National Strategy for Sustainable Low-Emission Cattle Farming, making it relevant to analyze whether these policies have considered the phenomenon of youth migration (Moreno et al., 2022; Díaz et al., 2024); and (iii) Guatemala and Colombia are the second and third countries receiving the most remittances as a result of migration, with US$18.11 billion and US$9.44 billion annually, respectively, only surpassed by Mexico with US$60.3 billion (Statista, 2023b).

The study aims to address two gaps in research, (i) although there is abundant literature on youth migration in some of the region’s countries, such as Mexico (Velasco, 2016; Suárez, 2023) and Brazil (Gaspar and Chatti, 2022; Spanevello et al., 2022), others have yet received less attention, for which the present study prioritizes two of the less-covered countries; and (ii) the migration topic has been primarily studied from its driving factors (Castelli, 2018; Barrios et al., 2022), gender perspectives (Everaert, 2021), or focus on population groups, such as youth (Cazzuffi and Fernández, 2018) (issues also addressed in this analysis), but is seldomly related to its effects on the cattle sector, which not only presents itself as a novelty in research but also proposes thematic lines for future investigations. Thus, this article addresses the following research question: what are the socio-economic drivers and impacts on the cattle sector of youth migration in Colombia and Guatemala, and how is the issue countered, if so, through the implementation of public policies?

The study is divided into the following sections: section 2 introduces fundamental concepts such as youth, migration, rurality, public policies, and laws, along with diverse theories of migration and the kaleidoscope model of policy change. Section 3 outlines the study’s characteristics, i.e., focus, stages, sources of information, and limitations. Section 4 identifies and analyzes the main drivers of migration, its effects, and implemented public policies. Section 5 provides methodological and practical recommendations and Section 6 formulates general conclusions.

## 2 Theoretical framework

To develop the stated objectives, it is necessary to clarify some concepts, such as *youth*, *rurality*, *laws*, and *public policies*. Additionally, reference is made to four theories of migration and the *Kaleidoscope Model of Policy Change*.

*Youth* refers to a diverse age range which is defined based on differing perspectives. They can be organized into two main groups, namely the national perspective, which is dependent on each country’s parameters, and the perspective of international organizations (Sandoval et al., 2022). In Colombia, a young person is anyone between 14 and 28 years of age, who is undergoing physical, intellectual, social, economic, and cultural development (Congreso de la República de Colombia, 2018). In Guatemala, the range is broader, involving individuals between 13 and 30 years old, divided into four groups: 13–17 years (adolescence), 18–20 years (late adolescence), 20–25 years (fully young adults), and 26–30 years (young adults) (Consejo Nacional de la Juventud y Fondo de Población de las Naciones Unidas, 2020). United Nations (2014) limit this concept to individuals between 15 and 24 years of age.

The term *rurality* often alludes to notions of the countryside, agriculture, and isolated, sparsely populated areas in contrast to the city, industry, and population density (Morales et al., 2015). The concept becomes more complex as current academic literature refers to a new rurality where the dichotomy is not as clear, and socio-economic dynamics possess characteristics of both scenarios. In this sense, the concept also encompasses spaces where non-agricultural activities and rural–urban relationships are strengthened (Chacón, 2021).

It is also important to distinguish between *laws* and *public policies*. While both aim to address various social issues, *laws* emerge from the congresses or parliaments of each country through specific constitutional procedures (Villadangos, 2022), while *public policies* are driven by national or local public institutions, either independently or in collaboration with non-governmental actors (private entities, NGOs, social groups, etc.) (Howlett and Cashore, 2014).

*Migration*, on the other hand, is defined as the movement of an individual from their place of residence to another, implying a substantial distance and resulting in permanent residence (Toney and Bailey, 2014). *Migration* can be within a region of the same country (internal) or beyond national borders (external), in which case it is termed immigration or emigration (Toney and Bailey, 2014).

On the other hand, the migration phenomenon can be theoretically explained by two variables: *push*, understood as factors that “push” a person to leave their place of residence, and *pull*, which are factors that attract, and both can be economic, social, political, environmental, and cultural, among others (Schiavon, 2023). The *push-pull theory* originated from the work of the German geographer Ernst Georg Ravenstein and was dominant in the first half of the 20th century, incorporating principles of political economy of the time, such as economic rationalism, individualism, and liberalism (García, 2018). The theory remains relevant in studies explaining migration from various perspectives, from migration between cities in China (Fangqu, 2022) to return migration in literature reviews (Mohamed and Abdul, 2020). However, the theory has also been criticized because it tends to list a series of factors that, while fostering migration, do not provide a structural and social explanation of the phenomenon (Hass, 2021), making it necessary to relate it to other theoretical postulates.

It is worth highlighting the *new economics of labor migration*, which states that (i) the decision to migrate is not individual but rather a risk management strategy of households and families in response to labor market failures, and (ii) there is the possibility that the migrant will return to their place of origin after achieving their goals of savings, investment, capital acquisition, and more (Gheasi and Nijkamp, 2017). It is also essential to refer to the *world-systems theory*, according to which migration is not a personal decision but the consequence of a structural dependency of poor countries on rich countries, as the latter have achieved higher levels of economic development and have the capacity and need to integrate labor (Morawska, 2021). Finally, the *migration systems theory* suggests that migration changes both micro factors (personal relationships) and macro factors (economic and social conditions) in the places of origin and destination, understanding the phenomenon in positive terms and in relation to development (Gheasi and Nijkamp, 2017).

To understand how governments have responded to the phenomenon of migration, particularly in the cattle sector, the *Kaleidoscope Model of Policy Change (KMPC)* can be applied. This model proposes a set of stages for analysis, namely (i) agenda-setting: determines topics of interest for governments; (ii) design: proposes solutions to the issue; (iii) adoption: considers adverse factors that may hinder policy implementation; (iv) implementation: executes the proposed solutions; and (v) evaluation and reform: establishes whether objectives were achieved or changes are necessary (Resnick et al., 2018). It is worth noting that this theoretical model has been applied in the analysis of nutrition policies in Africa (Hendriks et al., 2017) and fertilizer subsidies in Tanzania (Mather and Ndyetabula, 2016), demonstrating its adaptability to agricultural sector issues.

Considering these elements, it is necessary to make some clarifications. Firstly, in this study, *youth* include individuals aged between 13 and 30 years, allowing for the incorporation of both national and international definitions and encompassing a broader range of research on migration. Similarly, the definition of *new rurality* is invoked since in both Colombia and Guatemala, scenarios are identified that, while not corresponding to the traditional concept of rurality, involve phenomena of youth migration. It is noteworthy that the study simultaneously addresses *laws* and *public policies*, enabling a comprehensive analysis of legislative and governmental actions, while also referring to both internal and external migration.

**Figure 1:**
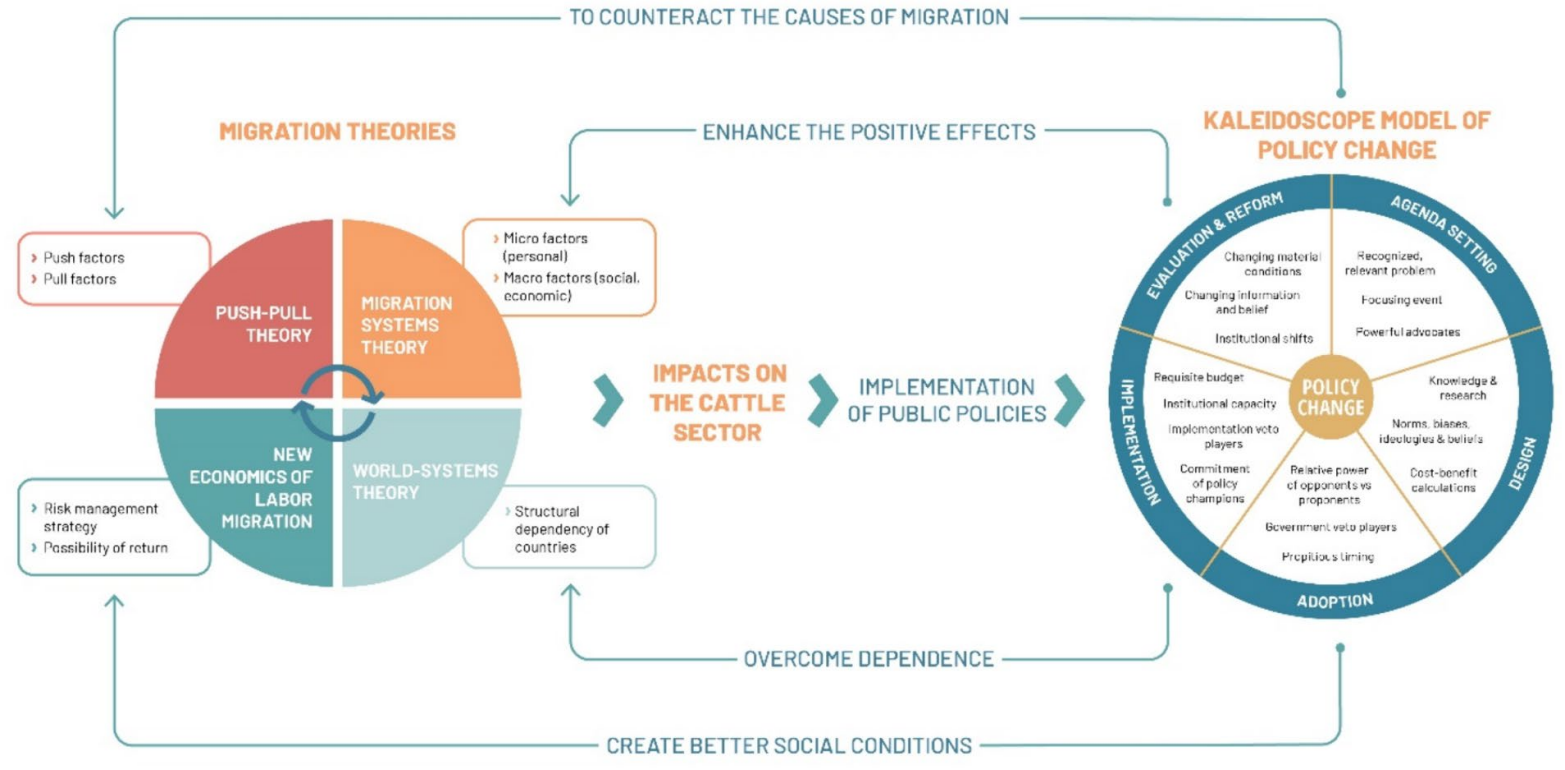
Theoretical framework of this study. Source: own elaboration.

Regarding the theories, although none directly address the migration-cattle relationship, their different postulates and interrelations construct a sufficiently broad analytical framework to understand the issue. For its part, the KMPC allows investigating whether public policies have responded to the migration phenomenon by counteracting its causes, creating better social conditions that encourage return, overcoming dependency on developed countries, and enhancing positive impacts (Figure 1).

## 3 Materials and methods

Based on the concept of systematic literature review (Palmatier et al., 2018), this study employed a critical analysis of documentary material regarding the phenomenon of youth migration in Colombia and Guatemala. For this purpose, a qualitative approach was applied, where concepts, characteristics, and descriptions were prioritized (Lune and Berg, 2017). The research followed a set of seven stages, namely (i) idea; (ii) formulation; (iii) definition of the study type; (iv) definition of information sources; (v) data collection; (vi) data analysis and discussion; and (vii) preparation of the final report (Sampieri et al., 2014). Secondary information sources were used (Sahu, 2013), divided into three types: (i) publications from state agencies, particularly public policies; (ii) documents from international organizations, such as FAO, OECD, USDA, providing accurate information on the addressed contexts; and (iii) academic articles to develop sections of the theoretical framework, results, analysis, and discussion. Data collection was carried out using the Google Scholar search engine, considering keywords such as *rural migration*, *youth migration*, *public policies*, and *cattle sector*. Two inclusion criteria were established, namely (i) publications from 2012 to 2022, to present the most up-to-date bibliography; and (ii) academic articles from indexed journals (Table 1).

**Table 1:** Sources of information.

Article section	Keywords	Country	Public entities	International organizations and statistical databases	Scientific articles
**4.1**	*Migration, youth, work, education, violence*	Colombia	Consejo Nacional de Política Económica y Social (CONPES) Departamento Administrativo Nacional de Estadística de Colombia (DANE) Unidad para las Víctimas	International Organization for Migration (IOM)	López et al. (2017) Mina and Téllez (2022) Torres (2021)
		Guatemala	**–**	Macrodata International Organization for Migration (IOM)	Santibáñez et al. (2017) Spohn (2017) Barrios et al. (2022) Everaert (2021) Jiménez (2016) Aguirre (2014) Lozano et al. (2015) Canales et al. (2016)
		Analysis	**–**	**–**	Cazzuffi and Fernández (2018) González et al. (2022)
**4.2**	*Migration, youth, cattle farming, labor, aging, modernization*	Colombia	DANE Ministerio de Agricultura y Desarrollo Rural (MADR)	Grupo Interagencial sobre Flujos Migratorios Mixtos (GIFMM)	Martínez et al. (2022) Aguilar and Serrano (2015) Triana and Burkart (2023)
		Guatemala	Gobierno de la República Ministerio de Agricultura, Pesca y Alimentación (MAPA) Consejo Agropecuario Centroamericano (CAC) Instituto Nacional de Estadística Guatemala (INE)	Economic Commission for Latin America and the Caribbean (ECLAC)	Villeda (2020) Weller (2016)
		Analysis	**–**	ECLAC	Gheasi and Nijkamp (2017) Groher et al. (2020) Morawska (2021)
**4.3**	*Public policy, youth, migration, unemployment, education, security*	Colombia	CONPES Congreso de la República MADR Proyectos Integrales de Desarrollo Agropecuario y Rural (PIDAR) Agencia de Desarrollo Rural (ADR) Ministerio de Educación Nacional (MEN) Servicio Nacional de Aprendizaje (SENA)	Food and Agriculture Organization of the United Nations (FAO)	Triana and Burkart (2023) Triana et al. (2020)
		Guatemala	Presidencia de la República Ministerio de Agricultura, Ganadería y Alimentación (MAGA) Ministerio de Trabajo y Prevision Social (MTPS) Consejo Nacional de Seguridad Consejo Nacional de la Juventud	United Nations Population Fund CEPAL Programa De Las Naciones Unidas Para El Desarrollo (PNUD)	**–**
		Analysis	**--**	**–**	García et al. (2019) Valenciano et al. (2016)

Source: own elaboration.

Among the study’s limitations, four points are noteworthy. First, there was a scarcity of literature addressing the issue of youth migration related to the cattle sector, leading to a reliance on the authors’ interpretations to a significant extent. Second, there are a vast number of public policies that directly and indirectly impact the addressed phenomenon, requiring the analysis to focus on national policies and exclude those at the local or regional levels. Third, due to the large number of public policies found, it was not possible to delve deeply into each one, limiting the analysis to a balance of results in each country. Fourth, since the study was limited to Colombia and Guatemala, generalizing the conclusions to the Latin American level is not possible.

## 4 Results, analysis, and discussion

### 4.1 Push-pull factors of migration

As discussed, migration involves push and pull factors and can occur internally or externally. The following factors are presented in the addressed scenarios, particularly concerning rural youth (aged 13–30 years).

In a study conducted in Arauca, Colombia, lack of employment was identified by Mina and Téllez (2022) as the main cause of youth migration, and youth lack sufficient support from public or private actors to start a business or formally enter the job market. The same authors indicate minimal technical-professional training for this population, coupled with excessive requirements for job access. A similar issue was identified by López et al. (2017) for Chinavita, Boyacá, where young migrants decide to leave primarily due to a lack of formal employment opportunities. In Guatemala, the search for better job opportunities is linked to variables such as the high cost of satisfying the basic family needs and widespread poverty (Santibáñez et al., 2017). Factors like low income, limited ability to acquire goods and services, unstable employment, and the responsibility of young people to financially support their parents are also evident (Santibáñez et al., 2017; Spohn, 2017).

The situation is particularly complex for women. In Guatemala, the main reasons for women not working in their place of origin are linked to a strong focus on unpaid household labor (79.1%) or receiving low salaries (16.5%) (Barrios et al., 2022). For girls and young women, internal migration from rural areas to urban centers is common, where they work as domestic workers, often receiving low, but some, payment (Santibáñez et al., 2017). In Colombia, rural women face a higher burden of unrecognized work, hindering their income generation (Consejo Nacional de Política Económica y Social, 2021). The lack of educational opportunities is also a significant factor, as seen in Guatemalan families migrating to the United States to provide better education for their young members (Spohn, 2017). A similar situation is observed in Colombia, where youth migration spans over generations, with parents stating they migrated as adolescents, just as their children do today (López et al., 2017).

In Colombia, another significant driver for migration is the internal armed conflict, a phenomenon that has affected the country since the 1950s, resulting in human rights violations (Mina and Téllez, 2022). The conflict has particularly affected young people between 18 and 28 years of age, with a national total of 2,172,373 victims accounted for over these decades, including victimizing events such as forced displacement, enforced disappearance, threats, kidnapping, land dispossession, among others (Unidad de Víctimas, 2017). While violence is not the primary factor for youth migration, it is mentioned by both women (10.4%) and men (3.1%) in Guatemala and the Northern Triangle (including El Salvador and Honduras) due to issues like extortion and, in the case of women, domestic violence (Everaert, 2021). In rural areas, the phenomenon of land appropriation by drug traffickers is strong, especially in border areas with little or no military or state presence, and farmers are offered an excessively low price for their land and are then forced to move (Jiménez, 2016). Land disputes also occur between farmers and landowners, with the latter taking advantage of the lack of property records or in alliance with state forces (Aguirre, 2014).

**Figure 2:**
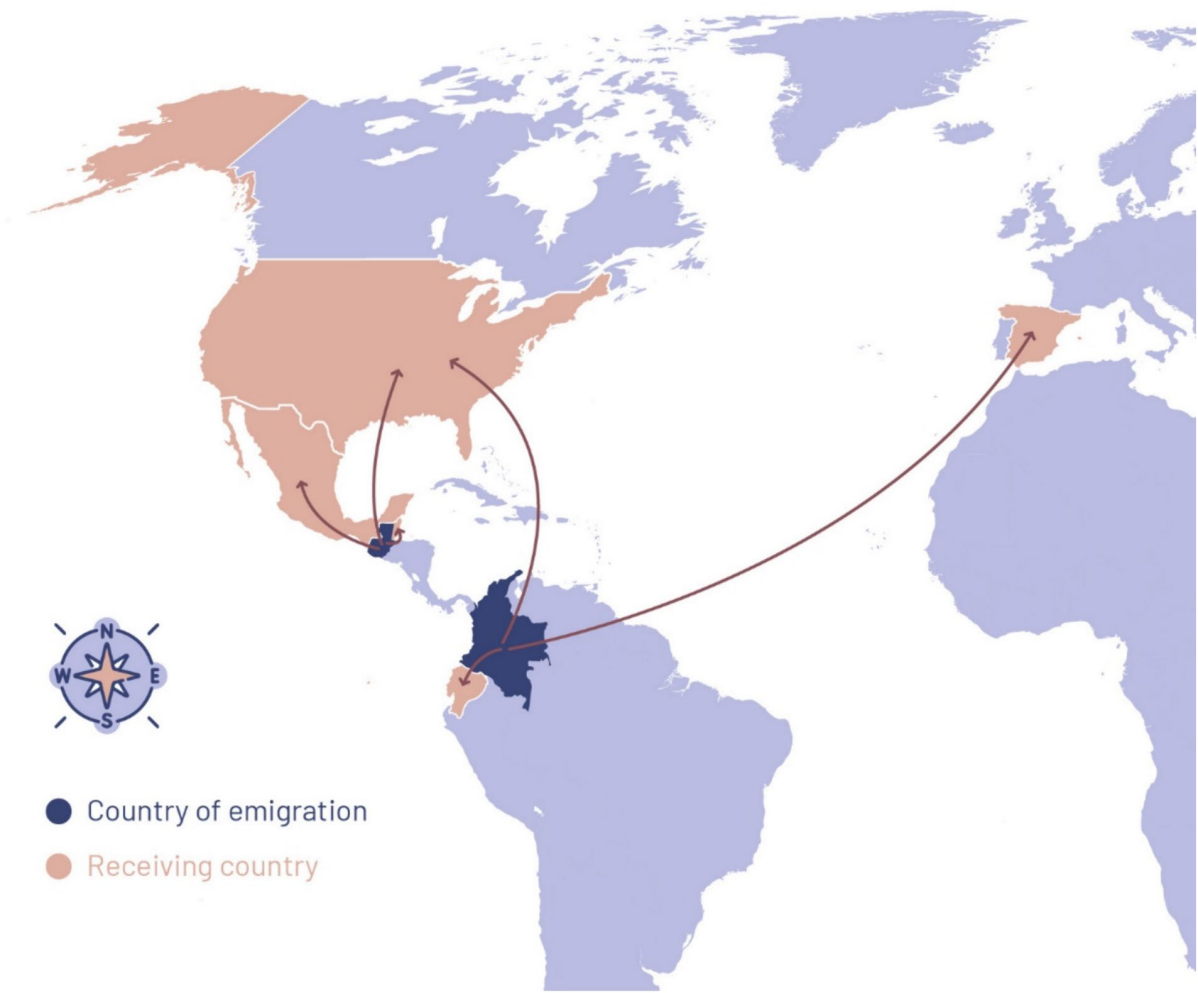
Map of migration from Colombia and Guatemala. Source: own elaboration.

Regarding the regions with the highest migration rates in Colombia, these include Bogotá (26.60%), Antioquia (7.30%), Cundinamarca (5.70%), and Valle del Cauca (5.60%), which are also the major recipients of migrants, with percentages of 14.90, 7.30, 17.20, and 6.40%, respectively (International Organization for Migration, 2021). These metropolitan areas represent pull factors for rural youth, such as salaries that allow them to increase their consumption and savings, as well as providing economic assistance to their relatives in their places of origin (Torres, 2021). They also concentrate the country’s main universities and offer protection from internal armed conflict dynamics (Mina and Téllez, 2022). External migration destinations include the United States (22%), Ecuador (20%), and Spain (14%) (DANE, 2022a; Figure 2). Although most migrants are adults (over 27 years old), young adults (19–26) and teenagers (12–18) represent 17.70 and 2.07% of them (International Organization for Migration, 2021). These destinations serve as pull factors for remittances, as the United States and Spain rank high in the origin of transfers to Colombia, especially to the departments of Valle del Cauca, Cundinamarca, and Antioquia (International Organization for Migration, 2021).

In Guatemala, internal migrants mainly head north in search of farmland or to Guatemala City, where more job opportunities exist (Lozano et al., 2015). Among the municipalities of origin are Santa Lucía La Reforma, Cabricán, and Zacualpa, where 75.86, 30.30, and 26.32% of migrants choose these destinations (Rocha and Gramajo, 2017). External migration happens mostly to the United States (89.65%), Mexico (3.38%), and Belize (1.97%) (Datos Macro, 2020). Notably, Guatemalan migrants are the youngest among the countries of the Northern Triangle, with 42% being under 24 years old and an average age of 27.7 years (Canales et al., 2016). The importance of external migration, particularly rural migration, is evident in the remittance figures, as this population benefits from 51.3% of remittances in regions such as San Marcos, Huehuetenango, Quetzaltenango, Quiché, and Escuintla (International Organization for Migration, 2022b).

In this regard, the presented elements reveal that youth migration is not driven by a single factor but is rather complex and multi-causal in both countries. Additionally, shortcomings of both public and private actors responsible for creating optimal conditions for study, work, and safety at the local, regional, and national levels are evident (as explored in detail in the third section). It is noteworthy that the addressed scenarios do not differ much from other Latin American contexts, as a study conducted in Ecuador, Mexico, and Peru found that the poles of expulsion are concentrated in rural areas, particularly those with high levels of poverty and dependence on the agricultural sector (Cazzuffi and Fernández, 2018). A similar situation exists in Spain, where rural depopulation occurred in past decades, and currently, there is a phenomenon of youth migration from small cities to capitals like Madrid and Barcelona (González et al., 2022). Migration is not only a problem but also has effects on economic sectors, necessitating the implementation of public policies for mitigation. This will be further addressed in the following sections.

Similarly, it is worth noting that, as proposed by the *new economics of labor migration* (Gheasi and Nijkamp, 2017), youth migration is not solely an individual decision but part of a collective decision by families seeking better opportunities, or they are those who play the role of “chosen ones” to migrate on behalf of the household and overcome market deficiencies such as lack of employment. It is also evident that, according to world-system theory (Morawska, 2021), Guatemala and Colombia fail to meet the economic and social needs of their citizens, leading to a dependency on labor and educational opportunities from developed countries like the United States, Spain, and even Mexico. As a positive factor, the inflow of remittances into disadvantaged rural areas allows families to improve their quality of life, an aspect that aligns with the concepts of migration systems theory by linking migration to the transformation of social and economic conditions (macro factors) in the place of origin, thereby fostering development (Gheasi and Nijkamp, 2017). In this regard, the opposition between theories is noteworthy, as such development entails a level of dependency on wealthy countries, as expressed earlier.

### 4.2 Effects of migration on the agricultural sector

As described in the previous section, certain factors drive young people to leave rural areas and migrate to urban areas or abroad. This phenomenon impacts various economic sectors, including agriculture, and particularly cattle farming in the targeted countries. The following discusses these impacts, along with some characteristics of the sector.

In Colombia, the agricultural sector employs 15.2% of young people aged between 15 and 28 years (DANE, 2023a). The average salary is COP766957 per month (~US$196) (DANE, 2022c), and informality reaches 85.4% (DANE, 2022d). It is noteworthy that departments with the highest cattle inventory, such as Antioquia (10.8%), Córdoba (8.3%), Casanare (8.2%), Meta (7.9%), Caquetá (6%), and Magdalena (5.7%) (DANE, 2022b), also present a high percentage of youth migration. Antioquia is one of the regions with the highest youth migration (7.3%), followed by Meta (3.1%), Córdoba (2.7%), Magdalena (2.4%), Caquetá (1.6%), and Casanare (1.3%) (International Organization for Migration, 2021).

**Figure 3:**
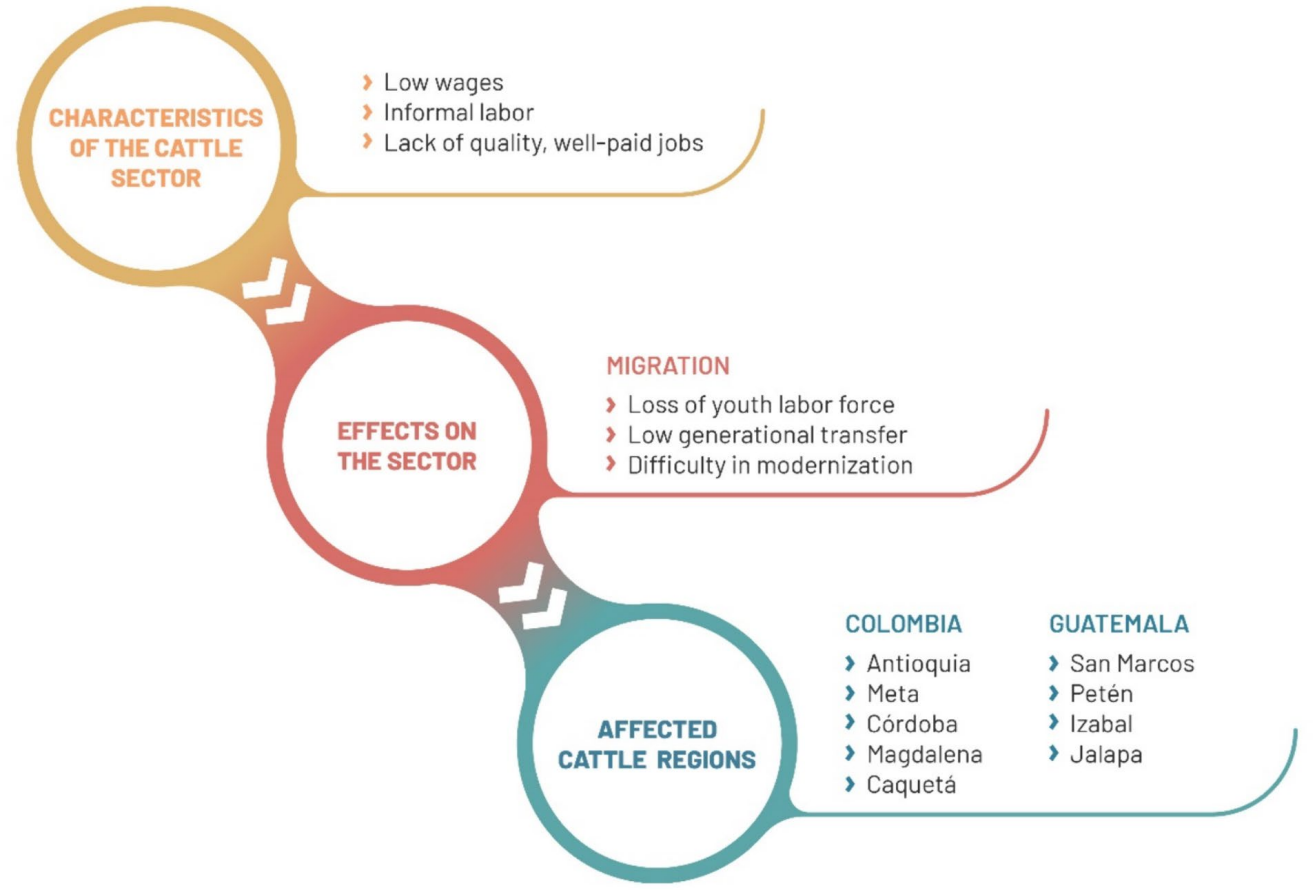
Causes and effects of youth migration in the cattle sector in Colombia and Guatemala. Source: own elaboration.

In these departments, and others with a focus on cattle farming, there is a loss of young labor, as seen in milk production systems in the Cañón de Anaime (Martínez et al., 2022). For producers, especially in medium-scale farms, retaining personnel for activities like milking is challenging (Ministerio de Agricultura y Desarrollo Rural, 2022a,b,c). Additionally, the sector demonstrates a low generational transfer, with an aging workforce and an inverted population pyramid (Ministerio de Agricultura y Desarrollo Rural, 2022a,b,c). According to the Third National Agricultural Census, the most recent and comprehensive statistic information at the national level, out of 725,225 cattle farmers living in rural areas (63.6% men and 36.4% women), the age group with the highest participation is individuals between 40 and 54 years old, constituting 32.7% (21.0% men and 11.7% women) (DANE, 2014). This situation negatively impacts the modernization of the sector, as older individuals lack sufficient knowledge of handling new technologies (Aguilar and Serrano, 2015) and often have a limited time horizon for long-term investments, i.e., in climate change adaptation and mitigation measures (Triana and Burkart, 2023; Figure 3).

In Guatemala, although the agricultural sector is the main source of employment, employing 35% of young people aged 15–29, it does not offer enough quality and well-paid jobs. The average salary is 1,155 quetzales per month (~US$150), and 90% of the employed population is informal (FAO, 2020). Petén, with approximately 54% of the cattle inventory, is the department with the highest cattle population (Ministerio de Agricultura, Pesca y Alimentación, 2023). In order of importance, it is followed by Izabal (also in the north of the country), Jalapa, Jutiapa, Santa Rosa (southeast), Retalhuleu, and San Marcos (southwest) (Gobierno de la República de Guatemala, 2018). It is worth noting that Petén has a low participation rate in youth migration at the national level (3.71%), but San Marcos is one of the departments with the highest rates (22.52%). Other agricultural regions with youth migration include Quiché (12.50%) and Quetzaltenango (6.29%) (Villeda, 2020), although these areas do not have a strong focus on cattle farming.

In a similar fashion to Colombia, youth migration in Guatemala, predominantly by men, leads to a reduced availability of rural labor (Consejo Agropecuario Centroamericano, 2022). This is compounded by an increasing need for labor in the construction and mining sectors, which offer more attractive salaries (Weller, 2016). However, 50% of young people aged 15–24 live in rural areas, indicating that generational turnover is not yet a problem (Comisión Económica para América Latina y el Caribe, 2019). On the contrary, cattle-focused departments such as Petén, Izabal, and San Marcos have average ages of 24.36, 26.05, and 25.54 years, respectively (Instituto Nacional de Estadística (INE), 2018). This circumstance is crucial for the modernization of the sector, especially considering cultural barriers to digital literacy in the adult population. For example, adults face challenges in understanding and using new technologies, and there is resistance to being instructed by children and young people on their use (Comisión Económica para América Latina y el Caribe, 2021).

In conclusion, youth migration emerges as an international issue, but the precarious labor conditions in local agricultural and livestock sectors in Colombia and Guatemala complicate the impacts, such as the availability of labor. In this regard, contrasting with the *migration systems theory* (Gheasi and Nijkamp, 2017), migration is not portrayed in positive terms linked to development, but rather as a phenomenon that threatens the livestock sector. Colombia’s response to this issue through Venezuelan migration is significant, considering the country’s hosting of 2,894,593 Venezuelan nationals. While the majority settle in urban areas (Grupo Interagencial sobre Flujos Migratorios Mixtos, 2023a), 345,739 are found in Antioquia alone (Grupo Interagencial sobre Flujos Migratorios Mixtos, 2023b), exemplifying the impact in cattle-focused regions and contributing to the labor force in this sector. However, the situation is different for Guatemala, as it is not a recipient country for migrants. Moreover, employing foreign labor does not provide a fundamental solution because it does not address the root causes of migration for the national population. Additionally, it is crucial to highlight that new technologies are an integral part of the cattle sector, including the use of robotics in animal husbandry or electronic data processing (Groher et al., 2020). Therefore, without a generational turnover capable of adapting to technological changes, these sectors in both countries will lag their international competitors. The above will affect job creation and the economy in general, perpetuating economic and social dependence on richer countries, as proposed by the *world-system theory* (Morawska, 2021).

### 4.3 Public policies to counteract youth migration

In the previous sections, the *push-pull* factors of youth migration were presented, along with their impacts on the agricultural, and particularly the cattle sector. Now, it is relevant to inquire about how governments have addressed this issue through the implementation of public policies and whether they have achieved the proposed results. To do this, it is pertinent to refer to the stages outlined by the KMPC.

**Figure 4:**
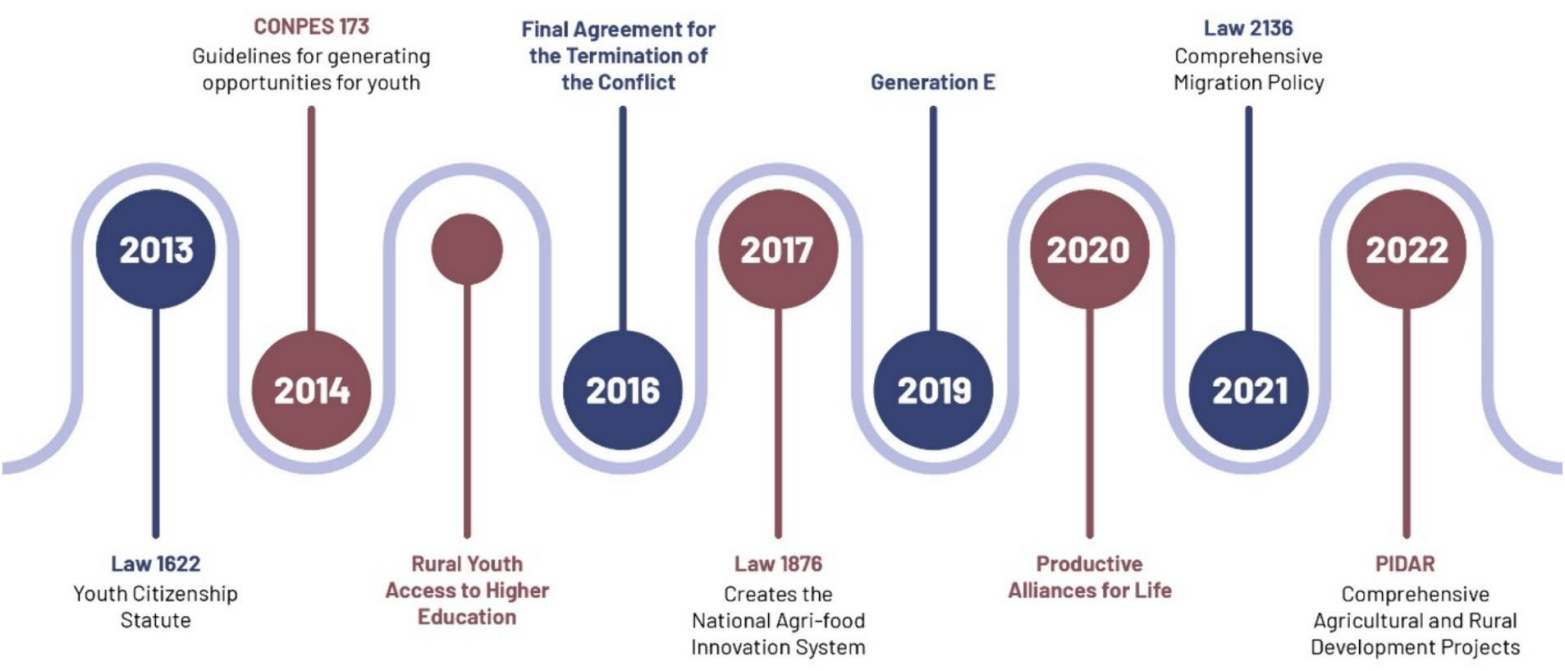
Timeline of public policies in Colombia. Source: own elaboration.

In Colombia, the *agenda setting* for public policies has determined topics of interest such as job creation, study opportunities, and security for rural youth. This has led to a *design phase* addressing these issues (Figure 4; Table 2). During the period under consideration, one noteworthy policy is the document CONPES 173 of 2014, which considers the principles of Law 1,622 of 2013, known as the Youth Citizenship Statute (Consejo Nacional de Política Económica y Social, 2014). Among its objectives, the document aims to strengthen access to productive entrepreneurship programs and provide opportunities for human development that facilitate generational turnover in rural areas (Consejo Nacional de Política Económica y Social, 2014). The Law 1876 of 2017 emphasizes the need to involve young people and women in agricultural production, enabling collective and efficient management of inputs, food, raw materials, and other resources (Congreso de la República de Colombia, 2017). It is also noteworthy to mention the Productive Alliances for Life, which aim to connect small producers with large markets, giving preference in its calls for proposals to projects composed of a minimum of 51% women and/or men aged between 18 and 28 years (Ministerio de Agricultura y Desarrollo Rural, 2022a,b,c). Projects like the Comprehensive Agriculture and Rural Development Projects (PIDAR) follow similar selection criteria, seeking to encourage income generation for rural residents and enhance the country’s competitiveness through the co-financing of projects (Agencia de Desarrollo Rural, 2022).

**Table 2:** Public policies to counteract youth migration in Colombia.

	Public policy	Promoting entity	Contribution of public policy
2013	Law 1,622. Youth Citizenship Statute (*Estatuto de Ciudadanía Juvenil*)	Congress of the Republic (*Congreso de la República*)	To ensure young people the full exercise of citizenship in civil, social, and public spheres
**2014**	CONPES 173. Guidelines for generating opportunities for youth *(Lineamientos para la generación de oportunidades para los jóvenes)*	National Council of Economic and Social Policy (*Consejo Nacional de Política Económica y Social*)	Develop strategies to ensure the transition of young people to working life in conditions of quality and stability
**2014**	Rural Youth Access to Higher Education *(Jóvenes Rurales Acceso a la Educación Superior)*	Ministry of Agriculture and Rural Development *(Ministerio de Agricultura y Desarrollo Rural)*	Improve access to rural education through credits and subsidies
**2016**	Final Agreement for the Termination of the Conflict *(Acuerdo Final para la Terminación del Conflicto)*	National Government; Revolutionary Armed Forces of Colombia (FARC-EP)*[Gobierno Nacional; Fuerzas Armadas Revolucionarias de Colombia – Ejército del Pueblo (FARC-EP)]*	End the internal armed conflict
**2017**	Law 1876. Creates the National Agri-food Innovation System *(Crea el Sistema Nacional de Innovación Agropecuaria)*	Congress of the Republic (*Congreso de la República*)	Ensure the participation of rural women and youth in processes offered by the National Agricultural Innovation System
**2019**	Generation E *(Generación E)*	National Government *(Gobierno Nacional)*	Increase access to quality higher education
**2020**	Productive Alliances for Life *(Alianzas Productivas para la Vida)*	Ministry of Agriculture and Rural Development *(Ministerio de Agricultura y Desarrollo Rural)*	Mitigate youth migration from rural areas to cities and strengthen income generation for young people and women
**2021**	Law 2,136. Comprehensive Migration Policy *(Política Integral Migratoria)*	Congress of the Republic *(Congreso de la República)*	Promote safe and orderly migration
**2022**	Comprehensive Agricultural and Rural Development Projects *[Proyectos Integrales de Desarrollo Agropecuario y Rural (PIDAR)]*	Rural Development Agency *(Agencia de Desarrollo Rural)*	Improve living conditions for rural residents, particularly women, youth, and conflict victims

Source: own elaboration.

Regarding the lack of study opportunities, the Rural Youth Access to Higher Education program was strengthened since the National Development Plan 2014–2018. This initiative provided credits and subsidies for academic program payments (Consejo Nacional de Política Económica y Social, 2021). The Generation E strategy also stands out, ensuring free enrollment for 200,000 young people in technical, technological, and university programs during its first 2 years of implementation (Ministerio de Educación Nacional, 2021). Among these, 63,000 students came from 719 municipalities considered rural or sparsely populated rural areas, and 54% of the beneficiaries were women (Consejo Nacional de Política Económica y Social, 2021). The promotion of agricultural and cattle technical education and double certification (academic-technical) in partnership with the National Learning Service (*Servicio Nacional de Aprendizaje*, SENA) has also been strengthened (FAO and SENA, 2021).

**Figure 5:**
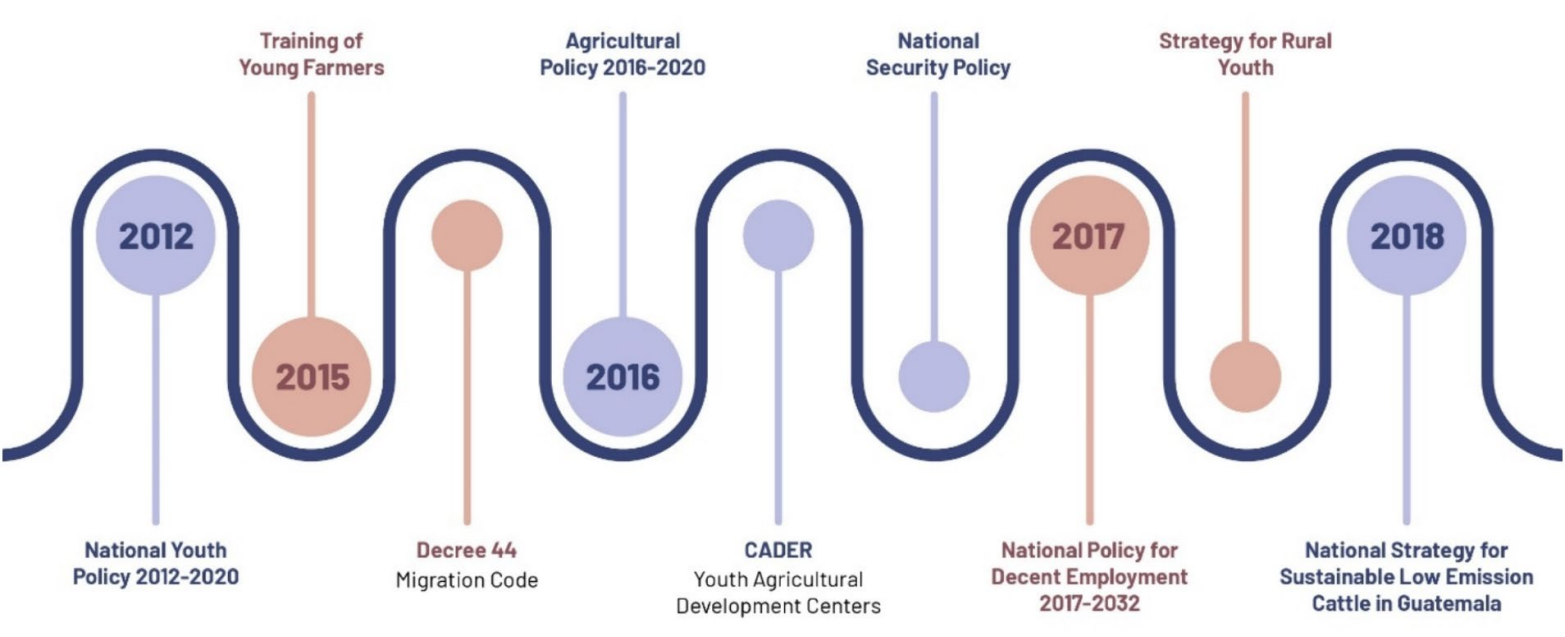
Timeline of public policies in Guatemala. Source: own elaboration.

Similarly, the *agenda setting* and *design* phases of public policies in Guatemala reveal a comprehensive set of training policies aimed at mitigating the unemployment of rural youth (Figure 5; Table 3). One of the most prominent is the National Youth Policy 2012–2020, which promotes training and job placement processes through a generational, gender, and regional approach (Presidencia de la República de Guatemala, 2012). This initiative is aligned with the Agricultural Policy 2016–2020, which recognizes the issue of youth migration while proposing a boost to new information technologies and income diversification to generate dignified employment for young people, attracting them to agricultural activities (Ministerio de Agricultura, Ganadería y Alimentación, 2016).

**Table 3:** Public policies to counteract youth migration in Guatemala.

Public policy		Promoting entity	Contribution of public policy
**2012**	National Youth Policy 2012–2020 *(Política Nacional de Juventud 2012–2020)*	Ministry of Social Development; National Youth Council; Planning and Programming Secretariat of the Presidency *(Ministerio de Desarrollo Social; Consejo Nacional de la Juventud; Secretaría de Planificación y Programación de la Presidencia)*	Improving the conditions and quality of life for young people by encouraging their integral development and citizenship exercise
**2015**	Training of Young Farmers *(Formación de jóvenes Agricultores)*	Helvetas (Swiss NGO); Ministry of Agriculture, Livestock, and Nutrition *[Ministerio de Agricultura, Ganadería y Alimentación (MAGA)]*	Training young people as entrepreneurs in the agricultural sector
**2016**	Decree 44. Migration Code *(Código de Migración)*	Congress of the Republic *(Congreso de la República)*	Recognizing the right of every person to migrate or emigrate
	Agricultural Policy 2016–2020 *(Política Agropecuaria 2016–2020)*	Ministry of Agriculture, Livestock, and Nutrition *[Ministerio de Agricultura, Ganadería y Alimentación (MAGA)]*	Strengthening the productivity of family farmers with an emphasis on young people and women
	Youth Agricultural Development Centers *(CADER Juveniles)*	Ministry of Agriculture, Livestock, and Nutrition *[Ministerio de Agricultura, Ganadería y Alimentación (MAGA)]*	Increasing the productivity of rural families
**2017**	National Security Policy *(Política Nacional de Seguridad)*	National Security Council *(Consejo Nacional de Seguridad)*	Proposing comprehensive actions for national security
	National Policy for Decent Employment 2017–2032 *(Política Nacional de Empleo Digno 2017–2032)*	Ministry of Labor and Social Welfare; International Labor Organization for Central America *(Ministerio de Trabajo y Previsión Social; Organización Internacional del Trabajo para América Central)*	Reducing poverty and inequality among young people and women
	Strategy for Rural Youth *(Estrategia para la Juventud Rural)*	Ministry of Agriculture, Livestock, and Nutrition *[Ministerio de Agricultura, Ganadería y Alimentación (MAGA)]*	Promoting equity and social inclusion for rural youth
**2018**	National Strategy for Sustainable Low Emission Cattle in Guatemala *(Estrategia Nacional de Ganadería Bovina Sostenible con Bajas Emisiones de Guatemala)*	Public sector; private sector; academia; NGOs; cattle sector organizations	Greater inclusion of young people and women in cattle organizations

Source: own elaboration.

With a greater emphasis on this population, the Rural Youth Strategy was formulated in 2017, among whose specific objectives is an offer of extension services that allow for the development of productive ventures (Ministerio de Agricultura, Ganadería y Alimentación, 2017). In the same year, the National Policy for Decent Employment 2017–2032 was presented, proposing the National Migration Program for Development as a priority action, fostering employment through the utilization of the “known how” of Guatemalan migrants with extended periods abroad (Ministerio de Trabajo y Previsión Social, 2017). Among other policies, it is essential to mention the Rural Learning Centers (CADER), driven by MAGA to transmit knowledge from a horizontal and practical approach in the quest for improving peasant economies (Asociación de Investigación y Estudios Sociales, 2022).

It is noteworthy that although violence is not one of the main push factors for migration, the Colombia’s Peace Agreement between FARC-EP and the government promotes the involvement of academic institutions in rural development. It also emphasizes promoting the productive permanence of young people in the field, beyond ending the armed confrontation between the State and the FARC-EP (Gobierno Nacional de Colombia y Fuerzas Armadas Revolucionarias de Colombia, 2016). In Guatemala, the National Security Policy proposes a local governance program where security is addressed jointly with other social needs, such as job creation (Consejo Nacional de Seguridad, 2017).

Other policies addressing various migration causes adhere to these policies. In the Decree 44 of 2016, Guatemala recognizes that its social and economic conditions generate a high number of migrants, necessitating the regulation of the phenomenon. Specifically for young people, it establishes the need for authorizations from parents for their departure, as well as compliance with the requirements of the receiving country (Congreso de la República de Guatemala, 2016). In Colombia, the Law 2,136 of 2021 acknowledges a diaspora of citizens abroad due to a multifactorial reality, aiming to promote orderly migration (Congreso de la República de Colombia, 2021). It establishes requirements such as the need for minors to have an exit permit from their father or mother, as stipulated by Article 110 of Law 1,098 of 2006 (Congreso de la República de Colombia, 2021).

Regarding policies promoted by the cattle sector, Guatemala’s Sustainable Cattle National Strategy proposes strengthening the inclusion of young people and women in productive and industrial organizations related to dairy and beef production. It also aims to increase the training of young technicians (Gobierno de la República de Guatemala, 2018). In contrast, Colombia’s Sustainable Cattle Policy, while referring to job creation, does not explicitly address this population (Ministerio de Agricultura y Desarrollo Rural, 2022a,b,c). In both scenarios, parallel to public policies, initiatives have been developed by the private sector. In Guatemala, it is crucial to refer to the Youth Farmers Agro-entrepreneurs Training Program [*Formación de Jóvenes Agricultores Agro-empresarios* (FORJA)], which prioritizes young people excluded from formal education systems (Ministerio de Agricultura, Ganadería y Alimentación, 2017). The program has been implemented in the departments of San Marcos, Huehuetenango, Alta Verapaz, and Quetzaltenango, training 985 young people, with 36% being women (Ministerio de Agricultura, Ganadería y Alimentación, 2017). In Colombia, the Heirs of Tradition program (*Herederos de Tradición*), driven by Alquería and SENA, aimed to engage rural youth in technological programs for cattle production. The program trained around 189 young people (60 women and 129 men) in five promotions, particularly in the departments of Meta, Cundinamarca, Nariño, Santander, and Cesar (Triana et al., 2020; Triana and Burkart, 2023).

**Figure 6:**
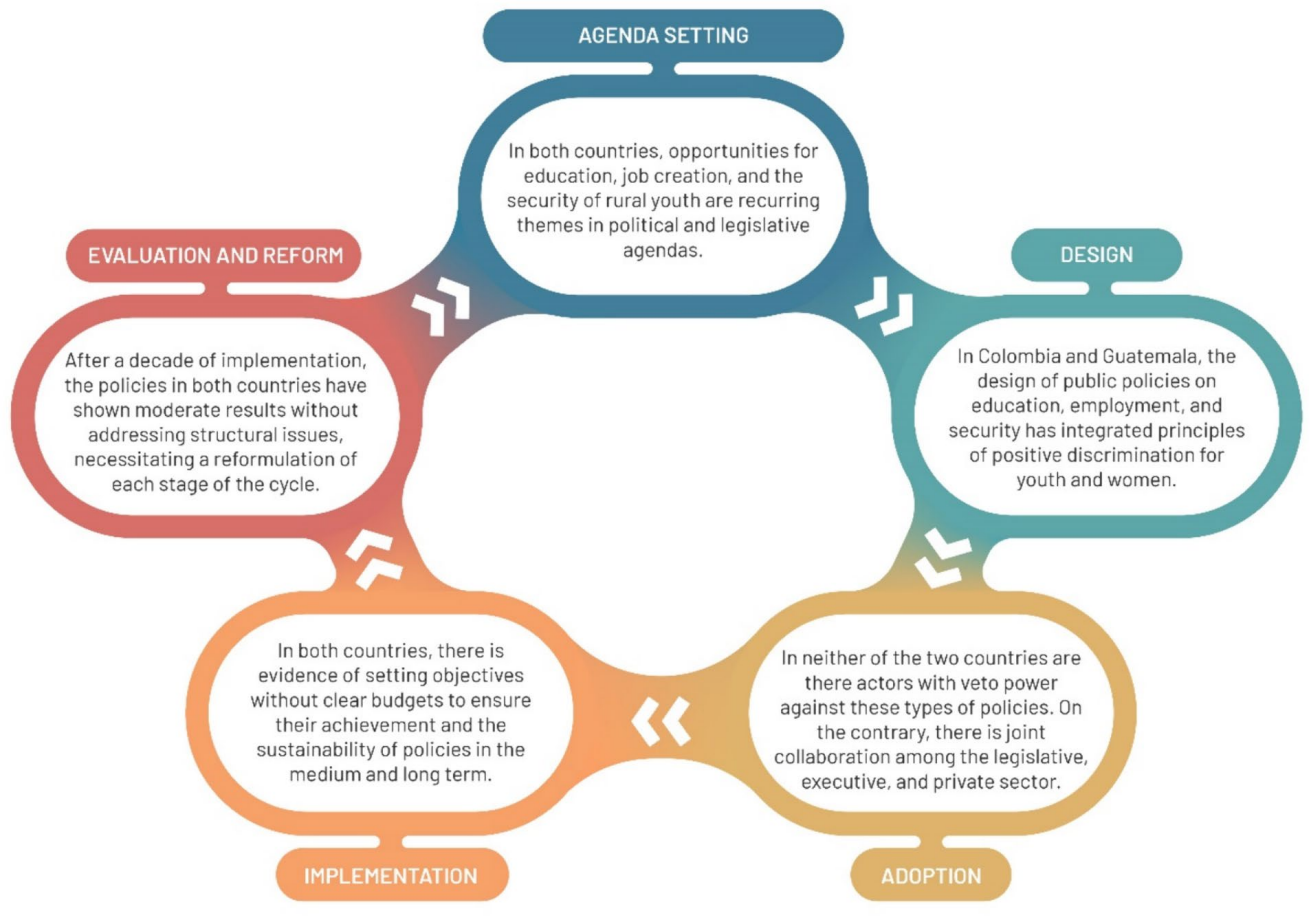
Application of the kaleidoscope model of policy change for Colombia and Guatemala. Source: own elaboration.

It is emphasized that the *adoption* of policies in both Colombia and Guatemala does not reveal opposition or veto players; on the contrary, there is a joint effort between the legislative and executive branches, involving multiple supportive actors, such as National Governments and Ministries, among others. However, the *implementation* of these policies shows an exposition of objectives without clear long-term budgets to ensure sustainability. Furthermore, after a decade of implementation, the *evaluation* is not entirely favorable (Figure 6).

In Guatemala, while urban youth aged 19–24 have an average of 9.10 years of education, rural youth in the same range only have 6.64 years (Consejo Nacional de la Juventud y Fondo de Población de las Naciones Unidas, 2020). Regarding gender, there are slight differences for the same age group. From the total population (48.3% men and 51.7% women), basic education was completed by 14.8% of men and 12.1% of women (Consejo Nacional de la Juventud y Fondo de Población de las Naciones Unidas, 2020). In Colombia, a stark contrast exists between urban and rural areas. While the average duration of study in urban areas is 9.2 years, in rural areas, it is only 5.5. Additionally, only two out of 10 rural high school graduates manage to enter higher education (Ministerio de Agricultura, Ganadería y Alimentación, 2017). However, unlike Guatemala, the percentage of rural youth (20–24 years) completing secondary education is higher in women (44.63%) than in men (38.64%) (Comisión Económica para América Latina y el Caribe, 2019).

Similarly, employment policies in Colombia reveal negative results, as the national youth unemployment rate (15–28 years) is 19% (DANE, 2023b). Unemployment rates are also unfavorable in cattle departments such as Córdoba (12.5%), Meta (11.2%), Caquetá (11.2%), and Magdalena (9.8%). These rates do not differ significantly from departments receiving migrants, and the latter even have higher figures, i.e., Antioquia (10.1%), Cundinamarca (12.7%), and Valle del Cauca (13.2%) (DANE, 2023c). In contrast, Guatemala has an unemployment rate of only 2.5%, but underemployment affects 9.4% of youth (15–24 years). Furthermore, 68% of the unemployed are under 30 years old, with those between 20 and 24 years being the most affected at 33.1% (Instituto de Investigaciones Económicas y Sociales, 2023). It is also noteworthy that the department of Guatemala has one of the highest percentages of wage employment at 60%, while agricultural and/or rural areas like Huehuetenango and Jalapa only have 34.9 and 35.1%, respectively (Programa de las Naciones Unidas para el Desarrollo, 2022).

These scenarios align with the situation in Mexico, where despite the objectives outlined in policies, they present partial results with moderate impact and no structural solutions (García et al., 2019). It is also worth noting that, while migration statutes contribute by regulating the phenomenon, in neither of the two countries of analysis do they aim to counteract its causes. Moreover, the Sustainable Cattle Policy in Colombia does not address rural youth employment, in contrast to the one in Guatemala. Similarly, while strategies initiated by the private sector are important, it should be the State leading employment and training programs in rural areas and not relinquishing this function. The pursuit of gender parity is a constant in the policies discussed, but results in this regard are not fully satisfactory, with men being the main participants and beneficiaries. This situation is not exclusive to Colombia and Guatemala but aligns with a Latin American context where legislative amendments and positive discrimination policies have not led to coherence between objectives and results, persisting gaps in access to credit, training, and technical assistance, among others (Valenciano et al., 2016).

Regarding the theories of migration addressed, several aspects stand out. Firstly, the failure of public policies and their inability to resolve the push factors of migration in Colombia and Guatemala have not allowed overcoming the structural dependence mentioned by the *world-system theory*. Instead, wealthy countries like the United States and Spain absorb their young workforce. Similarly, it is revealed that better social conditions and quality of life have not been generated to encourage the return of migrants, which is a possibility from the perspective of the *new economics of labor migration*. In contrast, migration continues to be a risk management strategy for young people and their families. Finally, besides the negative effects highlighted for the cattle sector, both countries lack mechanisms to maximize the positive impacts of migration mentioned by the *migration systems theory*, such as providing financial education to families to make effective use of remittances.

## 5 Conclusion and recommendations

Considering the research question, an evident conclusion unveils that unemployment, lack of study opportunities, and insecurity are the main drivers of rural youth migration in Colombia and Guatemala. This phenomenon has impacted the cattle sector by creating a shortage of labor and hindering generational transfer, thereby causing difficulties for the sector’s modernization, as well as implementing climate change adaptation and mitigation measures. Although multiple policies have been implemented to counteract these drivers, the results are negative, and the issue persists. In this regard, if the national governments in both countries do not adopt policies that go beyond partial responses, the cattle sector will lag behind its regional and international competitors, generating negative impacts on the achievement of the Sustainable Development Goals, i.e., SDG-1 (no poverty), SDG-2 (zero hunger), SDG-6 (gender equality), SDG-8 (decent work and economic growth), SDG-10 (reduced inequalities), SDG-13 (climate action), among others. These policies must continue while preserving their positive aspects, such as gender and youth perspectives, but with greater investments to reach a broader beneficiary population. Although this is a common consideration in public policy analysis, it is necessary to reiterate it because budgets are a crucial condition for achieving the stated objectives.

On the other hand, it is relevant to discuss the relevance of the theoretical models used and their potential contributions. The *push-pull theory of migration* is considered broad and flexible enough, allowing the exploration of a variety of variables (employment, education, security, etc.) and, thus, a comprehensive understanding of the phenomenon. It facilitates addressing migration in both its individual causes (unemployed youth) and structural causes (underfunded public policies). A theoretical contribution is seen in the proposed link between migration and public policies, as well as the impacts of the issue on a specific economic sector, such as cattle farming. Also highlighted is the contrast between the exposed negative effects of migration and the *migration systems theory*, suggesting a need to revise the positive conception of the phenomenon proposed by the theory. Regarding the *Kaleidoscope Model of Policy Change*, its capacity to understand public policies as a process that goes beyond setting objectives or executing solutions, requiring continuous evaluation, is reaffirmed. As a primary contribution to this theoretical model, its application in Latin American contexts stands out, as its antecedents are primarily in African countries. Likewise, it stands out that the lack of a specific theory to address the migration-cattle relationship was overcome by articulating various theoretical postulates, thus constructing an analytical framework that can be used in future research.

Among the methodological recommendations, there is a proposal to expand local-level studies addressing the links between youth migration and generational transfer, lack of labor, and modernization of the cattle sector, as research on this aspect is considered insufficient in the contexts addressed. Additionally, it is recommended to conduct comparative studies between developing countries (such as Colombia and Guatemala) and developed countries (such as the United States and European nations) with the aim of identifying successes, failures, and commonalities among implemented policies. Another possibility is the development of studies that contrast countries with and without internal armed conflict, which would allow identifying differences between migration due to violence and migration due to social or economic reasons.

In terms of practical recommendations, there is an emphasis on the need for Colombia’s Sustainable Cattle Policy to focus on social components for generating employment for youth and women. For both countries, the design of a policy that aims to counteract the issue rather than merely regulating migration is suggested. Finally, it is proposed to establish financial education programs for families receiving remittances so that they can develop microenterprises that promote employment and prevent future migration.

## Data availability statement

Data will be made available upon reasonable request.

## Author contributions

MD: Conceptualization, Formal analysis, Investigation, Methodology, Resources, Writing – original draft, Writing – review & editing. LM: Formal analysis, Resources, Writing – original draft, Writing – review & editing. SB: Conceptualization, Funding acquisition, Project administration, Resources, Supervision, Writing – original draft, Writing – review & editing. NT: Funding acquisition, Methodology, Project administration, Supervision, Writing – original draft, Writing – review & editing.
